# Broadly Neutralizing Antibody Characteristics in Hepatitis C Virus Infection and Implications for Vaccine Design

**DOI:** 10.3390/vaccines13060612

**Published:** 2025-06-06

**Authors:** Nicole E. Skinner

**Affiliations:** 1 The Abigail Wexner Research Institute at Nationwide Children’s Hospital, Columbus, OH 43205, USA; nicole.skinner@nationwidechildrens.org; 2 Department of Internal Medicine, College of Medicine, The Ohio State University, Columbus, OH 43210, USA

**Keywords:** hepatitis C virus, vaccine, broadly neutralizing antibody

## Abstract

Despite the use of direct-acting antiviral medications to treat hepatitis C virus (HCV), over a million people are newly infected each year, highlighting the need for a prophylactic vaccine. Due to the remarkable genetic diversity of HCV and its many immune evasion mechanisms, an effective vaccine will need to elicit broadly neutralizing antibodies (bNAb). In addition to providing evidence that a prophylactic HCV vaccine is feasible, this review provides an overview of known HCV bNAb targets, common antibody sequence features associated with broad neutralization, and mechanisms of immune escape. Ongoing knowledge gaps in the field and promising future directions are also discussed.

## 1. Introduction

Hepatitis C virus (HCV) is an enveloped, single-stranded RNA virus that infects liver cells [1]. First time infection with HCV results in chronic infection in the majority of individuals [2,3], with particularly high rates of chronic infection being associated with certain demographic features, such male sex, age over 25 years old, African descent, and infection with non-genotype 1 virus [4,5]. Over time, chronic infection promotes the development of hepatic cirrhosis, liver failure, and hepatocellular carcinoma. Individuals with HCV are also at increased risk for a wide variety of extrahepatic disease manifestations, including lymphoma, cryoglobulinemia, autoimmune diseases, metabolic dysfunction, and glomerulonephritis, among others [6].

HCV is primarily transmitted via contact with infected blood. Historically, most infections resulted from blood transfusion or non-sterile medical technique. Although these remain significant etiologies of HCV transmission in low-income countries today, screening of the blood supply and measures to ensure sterility in medical procedures have nearly eliminated these as sources of infection in high-incomes countries. Instead, infection in high-income countries, such as the United States (US), primarily results from injection drug use [7]. The rise of the opioid epidemic in the US has led to a rise in HCV incidence and resulted in younger adults bearing the largest disease burden [8,9]. In addition, approximately 5% of pregnancies in women with HCV result in perinatal transmission. The rise in HCV among women of childbearing age has led to a concomitant rise in the number of infants infected with HCV [10,11,12].

The development of a highly effective cure has been a vital breakthrough in the fight against HCV. Direct-acting antivirals (DAA) were first approved in the US in 2014. These agents are now able to cure HCV in over 95% of infected individuals with 8–12 weeks of oral therapy [13,14]. However, barriers to treatment uptake, including cost, accessibility, and HCV underdiagnosis have hampered the ability of these agents to reach the people who need them [15,16,17].

Approximately 57 million people are thought to be infected with HCV worldwide [18], including an estimated 1% of the US population [19]. Moreover, since most infections occur in people who inject drugs, a population known to be underrepresented in survey data and less likely to seek medical care where they might receive a diagnosis, the true prevalence is likely much higher. Only about half of people living with HCV in the US are aware of their diagnosis [16]. Globally, the number of people with HCV aware of their diagnosis falls to 20% [20]. Due to morbidity and mortality associated with chronic HCV and its high prevalence globally, the World Health Organization has established a goal of elimination, defined as a dramatic (90%) decrease in rates of HCV infection by 2030. The majority of nations, including the US, are not on track to achieve this goal, despite the use of DAA for over a decade [21].

A significant barrier to elimination is new infections that occur after treatment with DAA. DAA-mediated cure does not confer protection against subsequent reinfection, and rates of reinfection are high among people who continue to use injection drugs [22,23,24]. As rates of new HCV infections exceed cures [25], it is clear that an approach toward elimination that focuses only on treatment is unlikely to be successful. Development of a vaccine to be used in concert with expanded treatment efforts will be vital in achieving HCV elimination. Just as DAA were rationally designed based on breakthroughs in the understanding of HCV replication [26], development of a successful vaccine will likely require a nuanced understanding of the interplay between the host immune system and HCV. Although much has been discovered about effective HCV immunity, there are still important gaps in our knowledge. Future work aimed at filling these gaps will be critical and may require new avenues of research. For instance, since research on chimpanzees was banned in the United States, there has been no reproducible animal model for HCV, which has been a significant limitation in studying the biology of infection and testing vaccine candidates. Exciting work to develop animal models as well as investigations into controlled human infection studies are at the frontier of HCV vaccine research.

This review will describe what is known about humoral immune responses to HCV and give a detailed analysis of bNAb targets and characteristics. HCV mechanisms of bNAb-mediated immune evasion will also be discussed. This overview of bNAbs in HCV will be oriented toward the relevance of these findings for vaccine development, with discussions both of the the feasibility of developing an HCV vaccine and of the work still needed to achieve this important goal.

## 2. Evidence for HCV Vaccine Feasibility

Vaccination against viruses that cause chronic infection is typically more difficult than for self-resolving infections. In self-resolving infections, an effective immune response is naturally elicited by infection and the goal is to use attenuated virus, killed virus, or viral antigens to mimic infection while avoiding the morbidity and mortality of a true infection. In chronic infections, the immune response is ineffectual, resulting in inability to clear the virus. Consequently, the task of vaccine development is not merely to mimic a natural immune response, but to generate a different kind of immune response, one that will be protective. The difficulty of achieving this can be seen in vaccine development efforts against chronic pathogens as diverse as HIV, tuberculosis, and herpes simplex virus. However, there have also been successes: the hepatitis B virus vaccine, the herpes zoster virus vaccine, and the human papilloma virus vaccines, for example. There are features of the immune response to HCV, unusual in other chronic infections, that give particular hope that a vaccine against HCV is feasible.

A complex innate and adaptive immune response arises following infection with HCV. In 25–30% of individuals, this immune response mediates viral clearance. In the other 75–80% of people, the response is insufficient and chronic infection is established [2,3]. Immune responses that mediate spontaneous clearance provide a significant degree of protection against reinfection. Although individuals who have previously spontaneously cleared HCV can become reinfected, they spontaneously clear reinfection 80% of the time [27], and many individuals have cleared multiple distinct infections [28]. In addition, reinfections that clear are marked by lower peak viremia and a shorter duration of viremia compared to initial infections [29], kinetics indicative of memory recall responses.

These data illustrate two key features of the immune response to HCV that suggest a vaccine is feasible: (1) some HCV infections spontaneously resolve, and resolution is dependent on adaptive immune responses; and (2) protective memory responses mediate clearance of reinfection in most people. The fact that spontaneous, immune-mediated clearance of HCV occurs, and it is at least partially protective, sets it apart from many chronic infections and provides an opportunity to define correlates of natural protection, which can then inform vaccine design.

## 3. Immune Correlates of Protection

T cell responses are critical for spontaneous clearance of HCV. While there is a delay in the development of HCV-specific CD4^+^ and CD8^+^ T cells until 8–12 weeks post-infection, the appearance of these cells correlates with a sharp decline in viral load. In individuals who spontaneously clear infection, highly proliferative, multifunctional HCV-specific T cells are present through clearance. In persistent infection, there is progressive loss of CD4^+^ and CD8^+^ T cell breadth and function [30,31]. Clearance of infection elicits long-lived memory T cell populations that mediate spontaneous clearance of reinfection [32,33,34]. While much has been written about the importance and characteristics of T cell immunity (reviewed in more detail, for example, in [31]), a successful HCV vaccine will likely need to elicit protective bNAb responses as well.

Like T cell responses, humoral immune responses are also delayed, arising 10–12 weeks after infection and developing shortly after cellular responses [35,36]. Humoral immunity, particularly the development of neutralizing antibodies (NAb), is an important mediator of spontaneous clearance. Hypogammaglobulinemic patients have lower rates of spontaneous clearance and more severe disease [37,38]. While antibodies to both structural and nonstructural HCV proteins arise during acute infection regardless of outcome, the presence of NAb targeting surface viral envelope proteins is associated with spontaneous clearance [39,40]. Passive transfer of anti-HCV antibodies prevents transmission of HCV in humans [41,42,43]. Passive transfer of broadly neutralizing antibodies (bNAb), which are antibodies that neutralize diverse HCV strains, prevents infection with heterologous virus in mouse [44,45,46,47] and chimpanzee [48] animal models. Furthermore, the majority of individuals who clear reinfection have neutralizing antibody responses [27]. While bNAb can, and often do, arise in people who develop chronic infection, the timing of the bNAb response differs between individuals who clear infection and those who develop persistent infection. Clearance requires early development of bNAb responses, while chronic infection is often characterized by weak or absent NAb early in infection. When NAb arise in chronic HCV, they generally develop later in the course of infection [40,49,50,51,52].

## 4. Targets of bNAb in HCV

The HCV genome encodes a single polypeptide that is co- and post-translationally processed to form 10 mature proteins. These are grouped into the structural (Core, E1, E2) and nonstructural (p7, NS2, NS3, NS4A, NS4B, NS5A, NS5B) proteins. The nonstructural proteins are primarily involved in processing of the HCV polyprotein and viral replication [53]. Along with Core, the non-structural proteins are found within the HCV viral particle. Only E1 and E2 are present externally on the surface of the viruses, embedded in the lipid envelope, most readily available for antibody recognition. E1 and E2 heterodimerize to form an E1E2 complex [54,55]. E1E2 complexes exist as dimers on the surface of the virus [56]. The function of E1E2 is to mediate viral binding and internalization into target cells, and it is therefore the primary target of neutralizing antibodies. Cell entry is a complex process which involves binding of virus to multiple entry receptors, most notably the tetraspanin CD81, scavenger receptor class B type I (SR-BI), claudin-1, and occludin. Initially, apolipoproteins present in the HCV envelope bind to LDL, VLDL, and SR-BI present on hepatocytes. E1E2 then binds to CD81 and SR-BI [57]. It is the E2 component of the E1E2 dimer that directly contacts CD81 and SR-BI. E2 binding to CD81 results in a conformational change in E2 necessary for fusion of the viral and host membranes [58]. The tight junction proteins claudin and occludin aid in internalization. The role of E1 in viral entry is still not completely understood, but E1 has interactions with multiple host receptors such as claudin-1, claudin-6, and CD36, and is thought to facilitate fusion [57,58].

**Figure 1:**
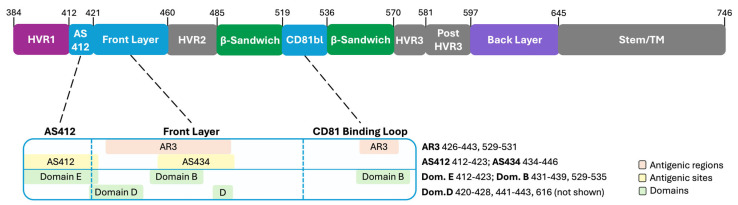
Schematic of the HCV E2 envelope glycoprotein. Above, regions within E2 are denoted, with the neutralizing face shown in blue (with the exception of amino acid 616 in the back layer). Amino acid numbering is shown at the top, using the H77 strain as a reference. Below, increased detail of regions within the neutralizing face of E2 is given, with amino acid numbering listed to the right [59,60,61].

E2, with its direct role in host receptor binding, has been long considered the main target of neutralizing antibodies to HCV, rather than E1. E2 is an approximately 360-amino-acid protein with a receptor-binding domain (RBD) linked to a transmembrane region by a stem region. The RBD has three variable regions (hypervariable region 1 [HVR1], HVR2, and HVR3), as well as a front layer, back layer, β-sandwich, and CD81 binding loop [55,58] (Figure 1). The stem and transmembrane domains are not primary targets of neutralizing antibodies, likely because they are not directly involved in interactions with host cells.

### 4.1. The Variable Regions of E2

HVR1 is a highly immunogenic, 27-amino-acid region at the N-terminus of E2. Although it is a major target for strain-specific NAb, its high variability and the absence of conserved residues means that HVR1-specific NAb are rarely broadly neutralizing [49,62,63]. HVR1 also rapidly acquires escape mutations to evade even narrow NAb responses. In addition, while many potential viral epitopes in E2 are heavily glycosylated, shielding them from recognition by antibodies, HVR1 is unobstructed by glycans, permitting antibody binding more readily [64]. For these reasons, HVR1 functions as a decoy region. Its immunodominance skews the antibody response toward highly mutagenic epitopes that readily escape immune pressure and away from more conserved regions of E2 [65,66,67,68]. In addition, antibodies bound to HVR1 shield these more conserved regions by sterically hindering bNAb binding. Thus, while HVR1 is not a primary target for bNAb, mutations in HVR1 can modulate the sensitivity of bNAb target regions to neutralization [69,70]. HVR2 and HVR3 form surface-exposed loops necessary for formation of heterodimers and enhance CD81 binding. Deletion of these regions abolishes viral entry. They are not significant targets for NAb, although like HVR1, they likely have effects on E2 structure that modulate sensitivity to bNAb targeting other regions, such as the CD81 binding loop [71,72].

### 4.2. The Neutralizing Face of E2

NAb targeting the remaining regions of the E2 RBD—the front layer, CD81 binding loop, β-sandwich, and back layer—have been identified. The front layer and CD81 binding loop are much more frequent targets of bNAb than the back layer or β-sandwich, and together comprise what is known as the neutralizing face of E2. The neutralizing face is a discontinuous region of amino acids that conformationally form a largely hydrophobic, surface-exposed portion of E2. Unlike the variable regions, the neutralizing face is highly conserved, overlapping with the CD81-binding site on E2 (amino acids 412–459, 519–536, and 616–617) [58,60]. The virus is restricted in its ability to mutate these regions without hindering its infectivity [73].

The neutralizing face is a hotspot for bNAb. Consequently, a number of neutralizing face bNAb have been identified [29,59], and the primary target regions within the neutralizing face have been characterized. There are two major, partially overlapping nomenclature systems used to reference bNAb target regions [59,74] (Figure 1). One system identifies five key antigenic domains (AD), all located within E2. The other system classifies continuous antigens, labeled as antigenic sites (AS), and discontinuous antigens, termed antigenic regions (AR). Within the neutralizing face, AS412/domain E spans amino acids 412–423 immediately downstream of HVR1. AS412 is highly conserved, with residues essential for CD81 binding [75]. Well-characterized bNAb HCV-1 [76], HC33.1 [77], and AP33 [78] target AS412. However, AS412 is not highly immunodominant, with only a minority of people generating bNAb to it during natural infection [60].

A key discontinuous antigenic region on the neutralizing face comprises amino acids 426–443 and 529–531, which are brought together by the conformational fold of E2 to form AR3. Overlapping with AR3 are domain B (amino acids 431–439 and 529–535) and domain D (amino acids 420–428 and 441–443 along with one back layer residue that forms part of the CD81-binding site, 616) (Figure 1). AR3-targeting antibodies include AR3A [47], HEPC3 [79], and HEPC74 [79], as well as HC-1 [77], HC-11 [77], and CBH-2 [80] targeting domain B, and HC84.26 [81] targeting domain D. AR3, and specifically domain B within AR3, is among the most immunodominant of bNAb targets [82]. A linear antigenic site in the front layer, AS434, spans amino acids 434–446 and therefore largely overlaps with domain D. AS434 forms a short, hydrophobic α-helix [60].

### 4.3. The β-Sandwich and the Back Layer of E2

AR1 and domain C are conformational antibody targets in the β-sandwich. Multiple antibodies have been isolated from patients with HCV targeting the β-sandwich, but they have generally not been broadly neutralizing. For instance, CBH-7 [77] is broadly reactive but weakly neutralizing. Similarly, AR1A [47], AR1B [47], and HEPC50 [79] are poorly neutralizing. AR2 and domain A are antigenic regions targeted by non-neutralizing antibodies within the back layer of E2, such as AR2A [47] and CBH-4B [77]. Thus, the β-sandwich and the back layer have historically not been thought to be targets for bNAb. Interestingly, however, a more recent study in which bNAb were isolated from an individual who resolved multiple HCV infections with broadly neutralizing plasma (termed an elite neutralizer) identified bNAb against both the back layer and the β-sandwich [83]. The crystal structure for one of the bNAb targeting the back layer showed that it recognized the AR4 antigenic region on the back layer, although in an E1-independent manner, unlike a previously identified AR4-binding bNAB, AR4A, described below [45].

### 4.4. E1 and the E1E2 Complex

In contrast to E2, much less is known about antibodies recognizing E1 or the E1E2 interface. This knowledge gap developed because of technical challenges in reproducing native folding of E1E2 or E1 alone. E1 does not appear to fold natively in the absence of E2. However, in 2022, the crystal structure of E1E2 was solved [55]. Around the same time, a soluble, natively-folded E1E2 containing a leucine zipper scaffold was produced [54]. From earlier work, two antigenic regions, AR4 and AR5 [45], were identified at the E1E2 interface. Although the E1E2 interface epitopes are not well-defined, they appear to be located near the C terminus of E2 and require E1 and E2 in complex for binding. Antibodies AR4A and AR5A recognizing these epitopes are both broadly neutralizing. The mechanism of neutralization in E1E2 antibodies is likely indirect. Rather than binding and blocking CD81 binding sites on E2, they are hypothesized to prevent an E1E2 conformational change necessary for viral entry.

Neutralizing E1 epitopes have also been identified. Antibody IGH526 and the related antibody IGH505 recognize an epitope near the C terminus of E1 (amino acids 314–324) [84]. They are potently cross-neutralizing. The structures of IGH526 and IGH505 in complex with E1E2 were solved by X-ray crystallography. The IGH526 epitope was found to be a discontinuous region within E1 with a linear component near the terminal membrane proximal region [85]. IGH505 targets a surface-exposed portion of a conserved α-helix in the E1 structure [55]. Based on their binding locations, the mechanism of neutralization for both antibodies was hypothesized to be inhibition of conformational changes in E1E2 necessary for viral entry. H-111 is a weakly-neutralizing antibody that recognizes a continuous epitope near the N terminus of E1 (amino acids 192–202) [86]. A narrowly neutralizing antibody, HEPC112, was found to recognize an epitope involving residues predominantly between amino acids 215 and 299 on E1, termed AS112 [87]. Interestingly, another antibody, A6, was identified that also targeted AS112 (amino acids 230–239) but was non-neutralizing [88]. There is still much to learn about the degree to which E1 is a target for bNAb and how E1-specific bNAb function.

## 5. Common Features of HCV-Specific bNAb

Just as there are common targets for bNAb on E1E2, there are commonalities in bNAb sequence and structure. The incredible diversity of B cell receptor (BCR) and antibody specificity derives in large part from recombination of heavy chain variable (V_H_), joining (J_H_), and diversity (D_H_) genes alongside recombination of light chain variable (V_L_) and joining (J_L_) genes. Humans have 51 V_H_ gene segments, 6 J_H_ segments, and 25 D_H_ segments from which one V_H_, D_H_, and J_H_ combine to form the heavy chains of a BCR. The heavy chains assemble with light chains, which can be either κ or λ light chains. For κ light chains, there are 50 V_κ_ and 5 J_κ_ gene segments. For λ light chains, there are 50 V_λ_ and 7 J_λ_ segments. In addition, random insertions and deletions of amino acids occur at the site of V(D)J joins. Lastly, somatic hypermutation occurring in germinal centers leads to additional mutation of the BCR in the variable gene. The combination of these mechanisms for diversity results in the theoretical possibility of generating up to 10^18^ unique B cell specificities [89]. Even when accounting for the fact that not all BCR will be functional, the potential diversity of the B cell repertoire is astronomical. Of course, the true number of unique B cell specificities is limited in reality by the total number of B cells within an organism. In the average human, there are about 10^11^ total B cells [90], approximately half of which are naïve [91]. The goal of rational HCV vaccine design is to identify which of these naïve B cell specificities, when expanded and affinity matured, provide the most potent and broadest protection against HCV, and then to determine which antigens best elicit expansion of these cells.

The dozens of bNAb identified from individuals with HCV, as well as a number of BCR repertoire analyses, provide insight into important antibody features associated with broad neutralization of HCV. One of the earliest commonalities observed in NAb isolated from individuals with HCV was preferential usage of the heavy chain gene V_H_1-69 [92]. V_H_1-69 is a fairly commonly expanded *IGHV* gene segment, present in only about 0.4% of naïve B cells, but used in about 4% of total B cells [93,94]. V_H_1-69 is preferentially used in many anti-viral bNAb, most notably bNAb against HIV, influenza, and HCV [92]. V_H_1-69 encodes two hydrophobic residues at the tip of the heavy chain complementarity determining region 2 (CDRH2) loop that can interact with hydrophobic regions of viral envelope proteins. Conserved hydrophobic residues are a feature of many viral envelope proteins because they facilitate fusion with the lipid membrane of host cells [95]. Both E1 and E2 have extensive hydrophobic regions, with hydrophobic interactions helping to stabilize their binding to one another as well. Importantly, as described above, the neutralizing face of E2 is largely hydrophobic [55]. Since most NAb isolated against HCV target the neutralizing face, a skewing toward V_H_1-69 usage is perhaps not surprising.

Further details regarding features of V_H_1-69 bNAb have emerged. V_H_1-69 is one of the most polymorphic of the *IGHV* genes, with 17 alleles. One of the key hydrophobic residues in CDRH2 is variant among these alleles. Ten alleles have a phenylalanine (F) at position 54, while seven have a leucine (L). Approximately 33% of people are homozygous for F-containing alleles, 11% are homozygous for L-containing alleles, and 56% are heterozygous. Across known anti-viral V_H_1-69 antibodies, including those against HCV, usage of F at position 54 is associated with broad neutralization, while usage of L is associated with weak or non-neutralization [92]. In addition, many front layer-specific V_H_1-69 bNAb have a longer than average CDRH3, often in conjunction with a stabilizing internal disulfide bond encoded by a D_H_2 diversity gene segment [96]. Disulfide-stabilized CDRH3 form β-hairpin motifs that directly interact with conserved front layer epitopes [96,97]. Another feature of V_H_1-69 bNAb that is reassuring for vaccine design is that most V_H_1-69 bNAb in HCV do not have high rates of somatic mutation [79,98]. Therefore, a great degree of affinity maturation is not required to produce HCV bNAb targeting the neutralizing face.

While early focus was on V_H_1-69 antibodies, it has become increasingly clear that not all HCV bNAb use V_H_1-69. For instance, in some studies in which antibodies were isolated from individuals who cleared HCV with broadly neutralizing plasma, bNAb that did not use V_H_1-69 were identified [79,87]. Most of these bNAb targeted sites in E1 or at the E1/E2 interface, but not all of them. BNAb HEPC154 targeted AR3 and used V_H_3-53 [87]. In an experiment using a machine learning algorithm to identify antibody features predictive of spontaneous clearance, V_H_4-39 and V_H_6-1 were identified as associated with spontaneous clearance [99]. Notably in that study, V_H_1-69 was more abundant in repertoires from chronically infected individuals than in patients who spontaneously cleared infection. In another study, RNA sequencing of the BCR was done in E2-reactive B cells from HCV-infected individuals with and without broadly neutralizing plasma. As expected, V_H_1-69 usage was highly enriched in individuals with broadly neutralizing plasma, present in >20% of E2-reactive B cell clonotypes. However, this approach also identified additional *IGHV* genes enriched in patients with bNAb responses (V_H_1-46, V_H_3-9, and V_H_3-64) [100]. In an analysis of a single individual with broadly neutralizing plasma who had spontaneously cleared multiple HCV infections, 55 monoclonal antibodies (mAb) were produced by sorting of E2-reactive B cells followed by sequencing of the BCR and cloning into immunoglobulin expression vectors. The mAb found to be broadly neutralizing demonstrated both expected commonalities and unexpected diversity. Using competition assays and structure analysis, bNAb were clustered based on E2 binding site. As described above, multiple bNAb targeting non-front layer regions of E2 were identified. BNAb targeting the front layer had certain amino acids enriched at positions in CDRH1, CDRH2, and CDRH3. These were not specific to one V gene, and many of them were found in crystal structure to be contact resides with E2. Five bNAb targeting the β-sandwich and five targeting the back layer were also identified. No bNAb targeting the β-sandwich and only one targeting the back layer used V_H_1-69, in contrast to six of nine front layer bNAb using V_H_1-69 [83]. Crystal structures of two front layer bNAb using V_H_1-46 were solved, which showed very similar binding epitopes and angles of approach as the canonical V_H_1-69 antibody HEPC74.

Together, these analyses demonstrate selection for V_H_1-69 usage among neutralizing face bNAb, likely due to the interaction between the hydrophobic tip of the CDRH2 and the hydrophobic neutralizing face. Long CDRH3, sometimes stabilized by a disulfide bond, and relatively low rates of somatic mutation also characterize these bNAb. More recent data is beginning to show that there are other paths to bNAb development as well. Even in the front layer, usage of V_H_1-46 may at times substitute for V_H_1-69, for example. BNAb can also target sites outside of the neutralizing face, where different *IGHV* genes and different binding characteristics are likely at play.

## 6. Immune Escape

One of the major challenges in HCV vaccine development is the virus’s considerable potential for escape from adaptive immune pressure. Understanding mechanisms of escape and how to circumvent them will be critical for vaccine design. HCV has developed a multitude of mechanisms for evading bNAb responses. The foremost of these is dramatic genetic diversity. NS5B, the HCV RNA polymerase, is error-prone and lacks proofreading ability. Error rates have been estimated to be between 10^−4^ and 10^−5^ substitutions per nucleotide per round of genome replication [101]. HCV is grouped into 8 distinct genotypes that differ from one another by approximately 30% of the genomic nucleotide sequence [101,102]. Within genotypes, there are 93 subtypes, with subtypes within a genotype differing from one another by an average of 15%. In addition, HCV replicates rapidly, resulting in the release of an estimated 10^12^ viral particles daily. Due to the high error rate combined with the high replication rate, it is estimated that every possible single mutation may be present within an infected individual. This intra-host variability results in so-called quasispecies which can differ from one another by up to 10% of nucleotides [101].

Substitutions in numerous amino acids conferring resistance to bNAb have been identified (reviewed in [59,61,97]). Because of its role in receptor binding, resistance mutations located within bNAb epitopes in the front layer of E2 often decrease viral fitness, although this is not always the case [103,104,105]. A large number of mutations that confer resistance to bNAb also occur outside of the targeted epitope. For instance, mutations in a region of the β-sandwich not known to be targeted by NAb confer resistance to multiple AR3/domain B bNAb [106]. As discussed above, multiple HVR1 mutations have also been found to contribute to bNAb resistance at distal sites [69,70]. Extra-epitopic resistance mutations likely function by inducing conformational changes in the targeted regions of E2 or by recruiting the binding of shielding non-neutralizing antibodies. Extra-epitopic resistance mutations that induce conformational changes can also negatively impact viral fitness [106,107].

Altering glycosylation is another key mechanism by which mutations within bNAb epitopes confer resistance. E1E2 is heavily glycosylated with up to five sites on E1 and eleven sites on E2, nine of which are highly conserved across HCV genotypes. Modifying N-linked glycosylation sites can result in glycan shielding, whereby glycans cover bNAb epitopes, making them inaccessible [64]. For instance, in one study, up to five glycans on E2 significantly reduced susceptibility to neutralization. In another study, a glycosylation shift from N417 to N415 resulted in development of resistance to multiple bNAb [108]. Interestingly, alterations in glycosylation can influence bNAb binding occurring at sites distal to the substitutions. Using an HCV cell culture model, Prentoe et al. found that removing glycans from E1E2 improved sensitivity to multiple bNAb even when the glycans were not within the bNAb epitopes. Surprisingly, deletion of HVR1 resulted in increased susceptibility to neutralization that was no longer modifiable by removal of glycans. Both removal of HVR1 and removal of glycans was found to shift E1E2 toward a less stable “open” conformation, resulting in decreased CD81 binding and increased susceptibility to bNAb [109].

It is also important to note that HCV has other mechanisms for antibody escape that do not directly relate to mutations within E1E2. For instance, HCV circulates in vivo as a complex of viral particles and host lipoproteins and apolipoproteins, forming lipoviral particles (LVP). When it exists as LVP, HCV has increased infectivity due to interactions with lipoprotein receptors in host cells and increased resistance to neutralization [110]. As one example, apolipoprotein E binding to HCV has been found to result in shielding of neutralizing epitopes on E2 [111]. In addition, in vitro HCV cell culture models suggest that cell-to-cell transmission of HCV independent of CD81 can occur [112,113]. Although it is not known the extent to which this occurs in vivo, this mechanism would be another way that HCV could bypass neutralizing antibodies.

## 7. HCV Vaccine Efforts

Vaccines can generally be classified into three groups: prophylactic vaccines that achieve sterilizing immunity (i.e., those that prevent development of infection), prophylactic vaccines that permit infection but lead to clearance of infection, and therapeutic vaccines that treat infection. Given the existence of highly effective DAA therapy for HCV, there is minimal interest in developing a therapeutic vaccine. A vaccine that elicits sterilizing immunity may be impossible to generate for a virus with as much variability as HCV. Even immunity conferred during cleared natural infection does not provide sterilizing immunity, but rather precipitates clearance of acute reinfection [27,28].

Consequently, a vaccine preventing chronic infection is an attractive goal [114,115]. There are a few downsides to this approach, however. A vaccine permitting acute infection would, by definition, not protect against morbidity or mortality caused by acute infection. In the case of HCV however, acute infections are usually asymptomatic [116,117], and fulminant liver failure is rare [118]. Most of the morbidity and mortality associated with HCV occurs during chronic infection. Secondly, a vaccine permitting acute infection allows individuals to transmit infection prior to clearance. However, a vaccine permitting acute infection would likely still decrease peak viral load and duration of viremia, as happens in reinfection after spontaneous clearance [29]. Lower viral loads and shorter periods of viremia may translate into decreased transmission rates. Therefore, despite these caveats, the greater feasibility of developing a non-sterilizing prophylactic vaccine makes it an appealing path forward.

The first and only clinical trial to test a vaccine preventing chronic infection in humans was done in a cohort of people who inject drugs [119], individuals who are at particularly high risk of infection with HCV. Subjects had never been infected with HCV at the time of vaccination and were followed after vaccination for infection and development of chronic HCV. The vaccine strategy was a heterologous prime boost approach. The prime was a chimpanzee adenovirus 3 vector engineered to express a portion of the nonstructural HCV genome (NS3-NS5B), with an inactivating mutation introduced into the catalytic site of the HCV polymerase, preventing replication. The boost was a Modified Vaccinia Virus Ankara vector containing the same insert as the prime. T cell responses were detected in 78% of vaccine recipients and viral loads during acute HCV were lower in vaccinated individuals, but rates of chronic HCV did not differ between subjects who received the vaccine and those who received placebo [119]. Although T cell responses are indispensable for spontaneous clearance of HCV, and some individuals are able to clear infection without a substantial bNAb response, this trial indicates that in a vaccination setting, eliciting only T cell responses without bNAb is likely not a viable approach.

A multitude of HCV vaccine candidates using a wide variety of platforms (including protein, viral vectors, DNA, mRNA, whole virus, and virus-like particles) are currently being investigated, as has been extensively reviewed elsewhere [120,121]. A significant challenge in evaluating vaccine candidates is the absence of an animal model that naturally recapitulates human infection. Antibody responses to vaccine candidates are often initially tested in the guinea pig or the mouse, neither of which are naturally infected with HCV and both of which have functional differences in their immune responses. For instance, the most variable portion of an antibody, the heavy chain CDRH3 is different in mice and humans. As discussed above, many HCV bNAb contain a longer than average CDRH3 and many also have a disulfide bond. Mouse CDRH3 are shorter than those in humans and only rarely participate in disulfide bond formation. They are also less diverse and have differences in amino acid composition [122]. Guinea pigs have almost twice as many heavy chain V gene (*IGHV*) alleles as humans, but notably do not have an analog of the human *IGHV* gene V_H_1-69 [123]. Historically, the chimpanzee model was used since chimpanzees are genetically similar to humans and are the only other animal naturally infected with HCV. However, the use of chimpanzees in research has been prohibited for ethical reasons. In addition, HCV infection in chimpanzees differs from infection in humans in a number of ways, notably that HCV is cleared more effectively in chimpanzees [124,125] and clearance occurs without NAb responses [126,127]. While macaques can be used to test immune responses to vaccination, they are not susceptible to infection with HCV, so cannot be challenged [125].

Given the limitations of animal models, there is growing enthusiasm in the field to establish a controlled human infection model (CHIM) [128,129]. Due to the availability of DAA, which are highly effective at eliciting virologic cure of HCV, it is now possible to infect humans with HCV experimentally and then cure them afterward. The only clinical trial to assess an HCV vaccine for prevention of chronic infection [119] took many years and much expense to complete due to the difficulty of recruiting individuals at risk for HCV and following them closely over time for development of infection. Use of a CHIM model would dramatically expedite the development of a vaccine by permitting efficient testing of candidates in humans. While the ethics of experimental human infection must be weighed carefully, CHIM has already been used successfully for studies on respiratory viruses, Dengue, Malaria, Cholera, and others [130]. The high benefit to society of a vaccine, the absence of alternative animal models for HCV, and the presence of curative HCV therapy support the development of CHIM in HCV.

## 8. Challenges and Future Directions

There are many challenges to the development of a prophylactic vaccine for HCV. The extensive diversity of the virus, greater even than that of HIV, makes development of a universal vaccine difficult. In addition, it will not be sufficient merely to generate an immune response to HCV, since both T and B cell responses are detected in individuals with chronic infection. Immunodominant, non-neutralizing antibody responses to HVR1 are promoted by the virus, for instance, but would ideally not be elicited by a vaccine as they are associated with persistent infection [68,131,132]. Developing a better understanding of correlates of protection is critical for vaccine design and for assessing vaccine candidate efficacy. For instance, must a vaccine elicit bNAb targeting multiple distinct epitopes on E1E2 to be broadly protective? And what are minimal protective thresholds for bNAb potency and breadth?

In addition, while this review has focused on bNAb responses, an effective vaccine will likely elicit both B and T cell responses, requiring a better understanding of how these arms of the immune response synergize in protective immune responses to HCV. Furthermore, although non-neutralizing antibody functions are important in many viral infections, little is known about what role they play in HCV infection. While a few studies have found evidence of antibody-dependent cellular cytoxicity and phagocytosis [133,134], more work is needed to determine whether these non-neutralizing mechanisms are important for antibody-mediated HCV clearance.

There is also significant uncertainty around optimal antigen selection for eliciting bNAb. Some vaccine candidates target E1E2 while others focus on E2 alone [120]. There is debate around whether to vaccinate with native antigens or to modify them, generating truncated and/or rationally designed constructs. Some advocate for removal of HVR1, given its role as decoy, while others have found that deletion of HVR1 may not affect overall vaccine immunogenicity [135]. Some groups are pursuing germline-targeting vaccines, which target specific, naturally occurring bNAb precursors, such as those encoded by V_H_1-69 [94]. Which HCV genotype and subtype the E2 sequence derives from may also have implications for the generalizability of the vaccine.

With so many possible vaccine candidates (noting that of course there are also a variety of vaccine platforms and potential adjuvants), having a means to stratify vaccine candidates so that only the most promising are taken to clinical trial will be important. In addition to understanding correlates of protection in humans, we will need to better understand how markers of protection do or do not translate to available animal models, such as the increasingly sophisticated rodent Hepacivirus models [136]. Ultimately, however, CHIM studies are likely to be groundbreaking in their capacity to accelerate our understanding of effective adaptive immunity to HCV and spur development of a prophylactic vaccine.

In sum, HCV represents a significant, global human health challenge. A highly effective cure, in the form of DAA, has been insufficient to stem the tide of new infections. Development of a prophylactic vaccine is imperative to achieve the WHO goal of elimination. For a vaccine to be successful, it is likely critical to elicit bNAb responses. We have gained great insights into the characteristics of bNAb responses to HCV, yet still have many important questions left to answer.

## Data Availability

No new data were created or analyzed in this study. Data sharing is not applicable to this article.

## References

[1] Purcell R. (1997). The hepatitis C virus: Overview. Hepatology.

[2] Lingala S., Ghany M.G. (2015). Natural history of hepatitis C. Gastroenterol. Clin. N. Am..

[3] Manns M.P., Buti M., Gane E., Pawlotsky J.-M., Razavi H., Terrault N., Younossi Z. (2017). Hepatitis C virus infection. Nat. Rev. Dis. Primers.

[4] Aisyah D.N., Shallcross L., Hully A.J., O’Brien A., Hayward A. (2018). Assessing hepatitis C spontaneous clearance and understanding associated factors—A systematic review and meta-analysis. J. Viral Hepat..

[5] Grebely J., Page K., Sacks-Davis R., van der Loeff M.S., Rice T.M., Bruneau J., Morris M.D., Hajarizadeh B., Amin J., Cox A.L. (2014). The effects of female sex, viral genotype, and IL28B genotype on spontaneous clearance of acute hepatitis C virus infection. Hepatology.

[6] Modaresi Esfeh J., Ansari-Gilani K. (2016). Steatosis and hepatitis C. Gastroenterol. Rep..

[7] Stroffolini T., Stroffolini G. (2024). Prevalence and modes of transmission of hepatitis C virus infection: A historical worldwide review. Viruses.

[8] Degenhardt L., Peacock A., Colledge S., Leung J., Grebely J., Vickerman P., Stone J., Cunningham E.B., Trickey A., Dumchev K. (2017). Global prevalence of injecting drug use and sociodemographic characteristics and prevalence of HIV, HBV, and HCV in people who inject drugs: A multistage systematic review. Lancet Glob. Health.

[9] Zibbell J.E., Asher A.K., Patel R.C., Kupronis B., Iqbal K., Ward J.W., Holtzman D. (2018). Increases in acute hepatitis C virus infection related to a growing opioid epidemic and associated injection drug use, United States, 2004 to 2014. Am. J. Public Health.

[10] Chen P.-H., Johnson L., Limketkai B.N., Jusuf E., Sun J., Kim B., Price J.C., Woreta T.A. (2023). Trends in the prevalence of hepatitis C infection during pregnancy and maternal-infant outcomes in the US, 1998 to 2018. JAMA Netw. Open.

[11] Mast E.E., Hwang L.-Y., Seto D.S., Nolte F.S., Nainan O.V., Wurtzel H., Alter M.J. (2005). Risk factors for perinatal transmission of hepatitis C virus (HCV) and the natural history of HCV infection acquired in infancy. J. Infect. Dis..

[12] Resti M., Azzari C., Mannelli F., Moriondo M., Novembre E., de Martino M., Vierucci A. (1998). Mother to child transmission of hepatitis C virus: Prospective study of risk factors and timing of infection in children born to women seronegative for HIV-1. BMJ.

[13] Alqahtani S.A., Sulkowski M.S. (2023). Chronic hepatitis C: Advances in Therapy and the remaining challenges. Med. Clin..

[14] Martinello M., Solomon S.S., Terrault N.A., Dore G.J. (2023). Hepatitis C. Lancet.

[15] Barber M.J., Gotham D., Khwairakpam G., Hill A. (2020). Price of a hepatitis C cure: Cost of production and current prices for direct-acting antivirals in 50 countries. J. Virus Erad..

[16] Kim H.s., Yang J.D., El-Serag H.B., Kanwal F. (2019). Awareness of chronic viral hepatitis in the United States: An update from the National Health and Nutrition Examination Survey. J. Viral Hepat..

[17] Terrault N.A. (2019). Hepatitis C elimination: Challenges with under-diagnosis and under-treatment. F1000Research.

[18] Blach S., Terrault N.A., Tacke F., Gamkrelidze I., Craxi A., Tanaka J., Waked I., Dore G.J., Abbas Z., Abdallah A.R. (2022). Global change in hepatitis C virus prevalence and cascade of care between 2015 and 2020: A modelling study. Lancet Gastroenterol. Hepatol..

[19] Hall E.W., Bradley H., Barker L.K., Lewis K., Shealey J., Valverde E., Sullivan P., Gupta N., Hofmeister M.G. (2024). Estimating hepatitis C prevalence in the United States, 2017–2020. Hepatology.

[20] World Health Organization (2021). Global Progress Report on HIV, Viral Hepatitis, and Sexually Transmitted Infections.

[21] Razavi H., Sanchez Gonzalez Y., Yuen C., Cornberg M. (2020). Global timing of hepatitis C virus elimination in high-income countries. Liver Int..

[22] Martinello M., Grebely J., Petoumenos K., Gane E., Hellard M., Shaw D., Sasadeusz J., Applegate T.L., Dore G.J., Matthews G.V. (2017). HCV reinfection incidence among individuals treated for recent infection. J. Viral Hepat..

[23] Midgard H., Bjøro B., Mæland A., Konopski Z., Kileng H., Damås J.K., Paulsen J., Heggelund L., Sandvei P.K., Ringstad J.O. (2016). Hepatitis C reinfection after sustained virological response. J. Hepatol..

[24] Valencia J., Alvaro-Meca A., Troya J., Cuevas G., Gutiérrez J., Morro A., Alvarez J., Pulido L., Cañamares I., Escobar I. (2019). High rates of early HCV reinfection after DAA treatment in people with recent drug use attended at mobile harm reduction units. Int. J. Drug Policy.

[25] Hill A.M., Nath S., Simmons B. (2017). The road to elimination of hepatitis C: Analysis of cures versus new infections in 91 countries. J. Virus Erad..

[26] Jesudian A.B., Gambarin-Gelwan M., Jacobson I.M. (2012). Advances in the treatment of hepatitis C virus infection. Gastroenterol. Hepatol..

[27] Osburn W.O., Fisher B.E., Dowd K.A., Urban G., Liu L., Ray S.C., Thomas D.L., Cox A.L. (2010). Spontaneous control of primary hepatitis C virus infection and immunity against persistent reinfection. Gastroenterology.

[28] Frumento N., Figueroa A., Wang T., Zahid M.N., Wang S., Massaccesi G., Stavrakis G., Crowe J.E., Flyak A.I., Ji H. (2022). Repeated exposure to heterologous hepatitis C viruses associates with enhanced neutralizing antibody breadth and potency. J. Clin. Investig..

[29] Kinchen V.J., Cox A.L., Bailey J.R. (2018). Can broadly neutralizing monoclonal antibodies lead to a hepatitis C virus vaccine?. Trends Microbiol..

[30] Smith S., Honegger J.R., Walker C. (2021). T-cell immunity against the hepatitis C virus: A persistent research priority in an era of highly effective therapy. Cold Spring Harb. Perspect. Med..

[31] Thimme R. (2021). T cell immunity to hepatitis C virus: Lessons for a prophylactic vaccine. J. Hepatol..

[32] Abdel-Hakeem M.S., Bédard N., Murphy D., Bruneau J., Shoukry N.H. (2014). Signatures of protective memory immune responses during hepatitis C virus reinfection. Gastroenterology.

[33] Nascimbeni M., Mizukoshi E., Bosmann M., Major M.E., Mihalik K., Rice C.M., Feinstone S.M., Rehermann B. (2003). Kinetics of CD4+ and CD8+ memory T-cell responses during hepatitis C virus rechallenge of previously recovered chimpanzees. J. Virol..

[34] Takaki A., Wiese M., Maertens G., Depla E., Seifert U., Liebetrau A., Miller J.L., Manns M.P., Rehermann B. (2000). Cellular immune responses persist and humoral responses decrease two decades after recovery from a single-source outbreak of hepatitis C. Nat. Med..

[35] Bertoletti A., Ferrari C. (2003). Kinetics of the immune response during HBV and HCV infection. Hepatology.

[36] Chen M., Sällberg M., Sönnerborg A., Weiland O., Mattsson L., Jin L., Birkett A., Peterson D., Milich D.R. (1999). Limited humoral immunity in hepatitis C virus infection. Gastroenterology.

[37] Christie J., Healey C., Watson J., Wong V., Duddridge M., Snowden N., Rosenberg W., Fleming K., Chapel H., Chapman R. (1997). Clinical outcome of hypogammaglobulinaemic patients following outbreak of acute hepatitis C: 2 year follow up. Clin. Exp. Immunol..

[38] Razvi S., Schneider L., Jonas M.M., Cunningham-Rundles C. (2001). Outcome of intravenous immunoglobulin-transmitted hepatitis C virus infection in primary immunodeficiency. Clin. Immunol..

[39] Lavillette D., Morice Y., Germanidis G., Donot P., Soulier A., Pagkalos E., Sakellariou G., Intrator L., Bartosch B., Pawlotsky J.-M. (2005). Human serum facilitates hepatitis C virus infection, and neutralizing responses inversely correlate with viral replication kinetics at the acute phase of hepatitis C virus infection. J. Virol..

[40] Pestka J.M., Zeisel M.B., Bläser E., Schürmann P., Bartosch B., Cosset F.-L., Patel A.H., Meisel H., Baumert J., Viazov S. (2007). Rapid induction of virus-neutralizing antibodies and viral clearance in a single-source outbreak of hepatitis C. Proc. Natl. Acad. Sci. USA.

[41] Feray C., Gigou M., Samuel D., Ducot B., Maisonneuve P., Reynes M., Bismuth A., Bismuth H. (1998). Incidence of hepatitis C in patients receiving different preparations of hepatitis B immunoglobulins after liver transplantation. Ann. Intern. Med..

[42] Knodell R., Ginsberg A., Conrad M., Bell C., Flannery E.P. (1976). Efficacy of prophylactic gamma-globulin in preventing non-A, non-B post-transfusion hepatitis. Lancet.

[43] Piazza M., Sagliocca L., Tosone G., Guadagnino V., Stazi M.A., Orlando R., Borgia G., Rosa D., Abrignani S., Palumbo F. (1997). Sexual transmission of the hepatitis C virus and efficacy of prophylaxis with intramuscular immune serum globulin: A randomized controlled trial. Arch. Intern. Med..

[44] De Jong Y.P., Dorner M., Mommersteeg M.C., Xiao J.W., Balazs A.B., Robbins J.B., Winer B.Y., Gerges S., Vega K., Labitt R.N. (2014). Broadly neutralizing antibodies abrogate established hepatitis C virus infection. Sci. Transl. Med..

[45] Giang E., Dorner M., Prentoe J.C., Dreux M., Evans M.J., Bukh J., Rice C.M., Ploss A., Burton D.R., Law M. (2012). Human broadly neutralizing antibodies to the envelope glycoprotein complex of hepatitis C virus. Proc. Natl. Acad. Sci. USA.

[46] Keck Z.Y., Wang Y., Lau P., Lund G., Rangarajan S., Fauvelle C., Liao G.C., Holtsberg F.W., Warfield K.L., Aman M.J. (2016). Affinity maturation of a broadly neutralizing human monoclonal antibody that prevents acute hepatitis C virus infection in mice. Hepatology.

[47] Law M., Maruyama T., Lewis J., Giang E., Tarr A.W., Stamataki Z., Gastaminza P., Chisari F.V., Jones I.M., Fox R.I. (2008). Broadly neutralizing antibodies protect against hepatitis C virus quasispecies challenge. Nat. Med..

[48] Morin T.J., Broering T.J., Leav B.A., Blair B.M., Rowley K.J., Boucher E.N., Wang Y., Cheslock P.S., Knauber M., Olsen D.B. (2012). Human monoclonal antibody HCV1 effectively prevents and treats HCV infection in chimpanzees. PLoS Pathog..

[49] Dowd K.A., Netski D.M., Wang X.H., Cox A.L., Ray S.C. (2009). Selection pressure from neutralizing antibodies drives sequence evolution during acute infection with hepatitis C virus. Gastroenterology.

[50] Esteban-Riesco L., Depaulis F., Moreau A., Bacq Y., Dubois F., Goudeau A., Gaudy-Graffin C. (2013). Rapid and sustained autologous neutralizing response leading to early spontaneous recovery after HCV infection. Virology.

[51] Logvinoff C., Major M., Oldach D., Heyward S., Talal A., Balfe P., Feinstone S., Alter H., Rice C., McKeating J.A. (2004). Neutralizing antibody response during acute and chronic hepatitis C virus infection. Proc. Natl. Acad. Sci. USA.

[52] Osburn W.O., Snider A.E., Wells B.L., Latanich R., Bailey J.R., Thomas D.L., Cox A.L., Ray S.C. (2014). Clearance of hepatitis C infection is associated with the early appearance of broad neutralizing antibody responses. Hepatology.

[53] Li H.-C., Yang C.-H., Lo S.-Y. (2021). Hepatitis C viral replication complex. Viruses.

[54] Metcalf M.C., Janus B.M., Yin R., Wang R., Guest J.D., Pozharski E., Law M., Mariuzza R.A., Toth E.A., Pierce B.G. (2023). Structure of engineered hepatitis C virus E1E2 ectodomain in complex with neutralizing antibodies. Nat. Commun..

[55] Torrents de la Peña A., Sliepen K., Eshun-Wilson L., Newby M.L., Allen J.D., Zon I., Koekkoek S., Chumbe A., Crispin M., Schinkel J. (2022). Structure of the hepatitis C virus E1E2 glycoprotein complex. Science.

[56] Augestad E.H., Holmboe Olesen C., Grønberg C., Soerensen A., Velázquez-Moctezuma R., Fanalista M., Bukh J., Wang K., Gourdon P., Prentoe J. (2024). The hepatitis C virus envelope protein complex is a dimer of heterodimers. Nature.

[57] Colpitts C.C., Tsai P.-L., Zeisel M.B. (2020). Hepatitis C virus entry: An intriguingly complex and highly regulated process. Int. J. Mol. Sci..

[58] Kumar A., Hossain R.A., Yost S.A., Bu W., Wang Y., Dearborn A.D., Grakoui A., Cohen J.I., Marcotrigiano J. (2021). Structural insights into hepatitis C virus receptor binding and entry. Nature.

[59] Sevvana M., Keck Z., Foung S.K., Kuhn R.J. (2021). Structural perspectives on HCV humoral immune evasion mechanisms. Curr. Opin. Virol..

[60] Tzarum N., Wilson I.A., Law M. (2018). The neutralizing face of hepatitis C virus E2 envelope glycoprotein. Front. Immunol..

[61] Velázquez-Moctezuma R., Augestad E.H., Castelli M., Holmboe Olesen C., Clementi N., Clementi M., Mancini N., Prentoe J. (2021). Mechanisms of hepatitis C virus escape from vaccine-relevant neutralizing antibodies. Vaccines.

[62] Farci P., Bukh J., Purcell R.H. (1997). The quasispecies of hepatitis C virus and the host immune response. Springer Seminars in Immunopathology.

[63] Shimizu Y.K., Hijikata M., Iwamoto A., Alter H.J., Purcell R.H., Yoshikura H. (1994). Neutralizing antibodies against hepatitis C virus and the emergence of neutralization escape mutant viruses. J. Virol..

[64] Lavie M., Hanoulle X., Dubuisson J. (2018). Glycan shielding and modulation of hepatitis C virus neutralizing antibodies. Front. Immunol..

[65] Kato N., Sekiya H., Ootsuyama Y., Nakazawa T., Hijikata M., Ohkoshi S., Shimotohno K. (1993). Humoral immune response to hypervariable region 1 of the putative envelope glycoprotein (gp70) of hepatitis C virus. J. Virol..

[66] Keck Z.-Y., Fuerst T.R., Mariuzza R.A., Foung S.K. (2016). B Cell Responses and Control of HCV Infection. Hepatitis C Virus I: Cellular and Molecular Virology.

[67] Ray R., Meyer K., Banerjee A., Basu A., Coates S., Abrignani S., Houghton M., Frey S.E., Belshe R.B. (2010). Characterization of antibodies induced by vaccination with hepatitis C virus envelope glycoproteins. J. Infect. Dis..

[68] Ray S.C., Wang Y.-M., Laeyendecker O., Ticehurst J.R., Villano S.A., Thomas D.L. (1999). Acute hepatitis C virus structural gene sequences as predictors of persistent viremia: Hypervariable region 1 as a decoy. J. Virol..

[69] Keck Z.-y., Girard-Blanc C., Wang W., Lau P., Zuiani A., Rey F.A., Krey T., Diamond M.S., Foung S.K. (2016). Antibody response to hypervariable region 1 interferes with broadly neutralizing antibodies to hepatitis C virus. J. Virol..

[70] Prentoe J., Jensen T.B., Meuleman P., Serre S.B., Scheel T.K., Leroux-Roels G., Gottwein J.M., Bukh J. (2011). Hypervariable region 1 differentially impacts viability of hepatitis C virus strains of genotypes 1 to 6 and impairs virus neutralization. J. Virol..

[71] Alhammad Y., Gu J., Boo I., Harrison D., McCaffrey K., Vietheer P.T., Edwards S., Quinn C., Coulibaly F., Poumbourios P. (2015). Monoclonal antibodies directed toward the hepatitis C virus glycoprotein E2 detect antigenic differences modulated by the N-terminal hypervariable region 1 (HVR1), HVR2, and intergenotypic variable region. J. Virol..

[72] Drummer H.E. (2014). Challenges to the development of vaccines to hepatitis C virus that elicit neutralizing antibodies. Front. Microbiol..

[73] Kinchen V.J., Zahid M.N., Flyak A.I., Soliman M.G., Learn G.H., Wang S., Davidson E., Doranz B.J., Ray S.C., Cox A.L. (2018). Broadly neutralizing antibody mediated clearance of human hepatitis C virus infection. Cell Host Microbe.

[74] Law M. (2021). Antibody responses in hepatitis C infection. Cold Spring Harb. Perspect. Med..

[75] Owsianka A.M., Timms J.M., Tarr A.W., Brown R.J., Hickling T.P., Szwejk A., Bienkowska-Szewczyk K., Thomson B.J., Patel A.H., Ball J.K. (2006). Identification of conserved residues in the E2 envelope glycoprotein of the hepatitis C virus that are critical for CD81 binding. J. Virol..

[76] Broering T.J., Garrity K.A., Boatright N.K., Sloan S.E., Sandor F., Thomas W.D., Szabo G., Finberg R.W., Ambrosino D.M., Babcock G.J. (2009). Identification and characterization of broadly neutralizing human monoclonal antibodies directed against the E2 envelope glycoprotein of hepatitis C virus. J. Virol..

[77] Pierce B.G., Keck Z.-Y., Lau P., Fauvelle C., Gowthaman R., Baumert T.F., Fuerst T.R., Mariuzza R.A., Foung S.K. (2016). Global mapping of antibody recognition of the hepatitis C virus E2 glycoprotein: Implications for vaccine design. Proc. Natl. Acad. Sci. USA.

[78] Owsianka A., Tarr A.W., Juttla V.S., Lavillette D., Bartosch B., Cosset F.-L., Ball J.K., Patel A.H. (2005). Monoclonal antibody AP33 defines a broadly neutralizing epitope on the hepatitis C virus E2 envelope glycoprotein. J. Virol..

[79] Bailey J.R., Flyak A.I., Cohen V.J., Li H., Wasilewski L.N., Snider A.E., Wang S., Learn G.H., Kose N., Loerinc L. (2017). Broadly neutralizing antibodies with few somatic mutations and hepatitis C virus clearance. JCI Insight.

[80] Owsianka A.M., Tarr A.W., Keck Z.-Y., Li T.-K., Witteveldt J., Adair R., Foung S.K., Ball J.K., Patel A.H. (2008). Broadly neutralizing human monoclonal antibodies to the hepatitis C virus E2 glycoprotein. J. Gen. Virol..

[81] Keck Z.-y., Xia J., Wang Y., Wang W., Krey T., Prentoe J., Carlsen T., Li A.Y.-J., Patel A.H., Lemon S.M. (2012). Human monoclonal antibodies to a novel cluster of conformational epitopes on HCV E2 with resistance to neutralization escape in a genotype 2a isolate. PLoS Pathog..

[82] Brasher N.A., Eltahla A.A., Underwood A., Boo I., Rizzetto S., Walker M.R., Rodrigo C., Luciani F., Maher L., Drummer H.E. (2020). B cell immunodominance in primary hepatitis C virus infection. J. Hepatol..

[83] Ogega C.O., Skinner N.E., Schoenle M.V., Wilcox X.E., Frumento N., Wright D.A., Paul H.T., Sinnis-Bourozikas A., Clark K.E., Figueroa A. (2024). Convergent evolution and targeting of diverse E2 epitopes by human broadly neutralizing antibodies are associated with HCV clearance. Immunity.

[84] Meunier J.-C., Russell R.S., Goossens V., Priem S., Walter H., Depla E., Union A., Faulk K.N., Bukh J., Emerson S.U. (2008). Isolation and characterization of broadly neutralizing human monoclonal antibodies to the e1 glycoprotein of hepatitis C virus. J. Virol..

[85] Kong L., Kadam R.U., Giang E., Ruwona T.B., Nieusma T., Culhane J.C., Stanfield R.L., Dawson P.E., Wilson I.A., Law M. (2015). Structure of hepatitis C virus envelope glycoprotein E1 antigenic site 314–324 in complex with antibody IGH526. J. Mol. Biol..

[86] Keck Z.-Y., Sung V.M., Perkins S., Rowe J., Paul S., Liang T.J., Lai M.M., Foung S.K. (2004). Human monoclonal antibody to hepatitis C virus E1 glycoprotein that blocks virus attachment and viral infectivity. J. Virol..

[87] Colbert M.D., Flyak A.I., Ogega C.O., Kinchen V.J., Massaccesi G., Hernandez M., Davidson E., Doranz B.J., Cox A.L., Crowe J.E. (2019). Broadly Neutralizing Antibodies Targeting New Sites of Vulnerability in Hepatitis C Virus E1E2. J. Virol..

[88] Mesalam A.A., Desombere I., Farhoudi A., Van Houtte F., Verhoye L., Ball J., Dubuisson J., Foung S.K., Patel A.H., Persson M.A. (2018). Development and characterization of a human monoclonal antibody targeting the N-terminal region of hepatitis C virus envelope glycoprotein E1. Virology.

[89] Rees A.R. (2020). Understanding the human antibody repertoire. mAbs.

[90] Sender R., Weiss Y., Navon Y., Milo I., Azulay N., Keren L., Fuchs S., Ben-Zvi D., Noor E., Milo R. (2023). The total mass, number, and distribution of immune cells in the human body. Proc. Natl. Acad. Sci. USA.

[91] Frasca D., Diaz A., Romero M., Landin A.M., Blomberg B.B. (2011). Age effects on B cells and humoral immunity in humans. Ageing Res. Rev..

[92] Chen F., Tzarum N., Wilson I.A., Law M. (2019). VH1-69 antiviral broadly neutralizing antibodies: Genetics, structures, and relevance to rational vaccine design. Curr. Opin. Virol..

[93] Briney B., Inderbitzin A., Joyce C., Burton D.R. (2019). Commonality despite exceptional diversity in the baseline human antibody repertoire. Nature.

[94] Capella-Pujol J., de Gast M., Radić L., Zon I., Chumbe A., Koekkoek S., Olijhoek W., Schinkel J., van Gils M.J., Sanders R.W. (2023). Signatures of VH 1-69-derived hepatitis C virus neutralizing antibody precursors defined by binding to envelope glycoproteins. Nat. Commun..

[95] Harrison S.C. (2005). Mechanism of membrane fusion by viral envelope proteins. Adv. Virus Res..

[96] Flyak A.I., Ruiz S., Colbert M.D., Luong T., Crowe J.E., Bailey J.R., Bjorkman P.J. (2018). HCV broadly neutralizing antibodies use a CDRH3 disulfide motif to recognize an E2 glycoprotein site that can be targeted for vaccine design. Cell Host Microbe.

[97] Frumento N., Flyak A.I., Bailey J.R. (2021). Mechanisms of HCV resistance to broadly neutralizing antibodies. Curr. Opin. Virol..

[98] Tzarum N., Giang E., Kong L., He L.A.-O., Prentoe J.A.-O., Augestad E.A.-O., Hua Y., Castillo S.A.-O., Lauer G.A.-O., Bukh J.A.-O. (2012). Genetic and structural insights into broad neutralization of hepatitis C virus by human V(H)1-69 antibodies. Sci. Adv..

[99] Eliyahu S., Sharabi O., Elmedvi S., Timor R., Davidovich A., Vigneault F., Clouser C., Hope R., Nimer A., Braun M. (2018). Antibody repertoire analysis of hepatitis C virus infections identifies immune signatures associated with spontaneous clearance. Front. Immunol..

[100] Skinner N.E., Ogega C.O., Frumento N., Clark K.E., Yegnasubramanian S., Schuebel K., Meyers J., Gupta A., Wheelan S., Cox A.L. (2023). Convergent antibody responses are associated with broad neutralization of hepatitis C virus. Front. Immunol..

[101] Perales C. (2020). Quasispecies dynamics and clinical significance of hepatitis C virus (HCV) antiviral resistance. Int. J. Antimicrob. Agents.

[102] Borgia S.M., Hedskog C., Parhy B., Hyland R.H., Stamm L.M., Brainard D.M., Subramanian M.G., McHutchison J.G., Mo H., Svarovskaia E. (2018). Identification of a novel hepatitis C virus genotype from Punjab, India: Expanding classification of hepatitis C virus into 8 genotypes. J. Infect. Dis..

[103] Keck Z.-Y., Olson O., Gal-Tanamy M., Xia J., Patel A.H., Dreux M., Cosset F.-L.c., Lemon S.M., Foung S.K. (2008). A point mutation leading to hepatitis C virus escape from neutralization by a monoclonal antibody to a conserved conformational epitope. J. Virol..

[104] Keck Z.-Y., Saha A., Xia J., Wang Y., Lau P., Krey T., Rey F.A., Foung S.K. (2011). Mapping a region of hepatitis C virus E2 that is responsible for escape from neutralizing antibodies and a core CD81-binding region that does not tolerate neutralization escape mutations. J. Virol..

[105] Velázquez-Moctezuma R., Galli A., Law M., Bukh J., Prentoe J. (2019). Hepatitis C virus escape studies of human antibody AR3A reveal a high barrier to resistance and novel insights on viral antibody evasion mechanisms. J. Virol..

[106] Bailey J.R., Wasilewski L.N., Snider A.E., El-Diwany R., Osburn W.O., Keck Z., Foung S.K., Ray S.C. (2015). Naturally selected hepatitis C virus polymorphisms confer broad neutralizing antibody resistance. J. Clin. Investig..

[107] Keck Z.-y., Li S.H., Xia J., von Hahn T., Balfe P., McKeating J.A., Witteveldt J., Patel A.H., Alter H., Rice C.M. (2009). Mutations in hepatitis C virus E2 located outside the CD81 binding sites lead to escape from broadly neutralizing antibodies but compromise virus infectivity. J. Virol..

[108] Pantua H., Diao J., Ultsch M., Hazen M., Mathieu M., McCutcheon K., Takeda K., Date S., Cheung T.K., Phung Q. (2013). Glycan shifting on hepatitis C virus (HCV) E2 glycoprotein is a mechanism for escape from broadly neutralizing antibodies. J. Mol. Biol..

[109] Prentoe J., Velázquez-Moctezuma R., Augestad E.H., Galli A., Wang R., Law M., Alter H., Bukh J. (2019). Hypervariable region 1 and N-linked glycans of hepatitis C regulate virion neutralization by modulating envelope conformations. Proc. Natl. Acad. Sci. USA.

[110] Sidorkiewicz M. (2021). Hepatitis C virus uses host lipids to its own advantage. Metabolites.

[111] Fauvelle C., Felmlee D.J., Crouchet E., Lee J., Heydmann L., Lefèvre M., Magri A., Hiet M.-S., Fofana I., Habersetzer F. (2016). Apolipoprotein E mediates evasion from hepatitis C virus neutralizing antibodies. Gastroenterology.

[112] Brimacombe C.L., Grove J., Meredith L.W., Hu K., Syder A.J., Flores M.V., Timpe J.M., Krieger S.E., Baumert T.F., Tellinghuisen T.L. (2011). Neutralizing antibody-resistant hepatitis C virus cell-to-cell transmission. J. Virol..

[113] Timpe J.M., Stamataki Z., Jennings A., Hu K., Farquhar M.J., Harris H.J., Schwarz A., Desombere I., Roels G.L., Balfe P. (2008). Hepatitis C virus cell-cell transmission in hepatoma cells in the presence of neutralizing antibodies. Hepatology.

[114] Barnes E., Cooke G.S., Lauer G.M., Chung R.T. (2023). Implementation of a controlled human infection model for evaluation of HCV vaccine candidates. Hepatology.

[115] Walker C.M. (2017). Designing an HCV vaccine: A unique convergence of prevention and therapy?. Curr. Opin. Virol..

[116] Cox A.L., Netski D.M., Mosbruger T., Sherman S.G., Strathdee S., Ompad D., Vlahov D., Chien D., Shyamala V., Ray S.C. (2005). Prospective evaluation of community-acquired acute-phase hepatitis C virus infection. Clin. Infect. Dis..

[117] Westbrook R.H., Dusheiko G. (2014). Natural history of hepatitis C. J. Hepatol..

[118] Kamal S.M. (2008). Acute hepatitis C: A systematic review. Off. J. Am. Coll. Gastroenterol..

[119] Page K., Melia M.T., Veenhuis R.T., Winter M., Rousseau K.E., Massaccesi G., Osburn W.O., Forman M., Thomas E., Thornton K. (2021). Randomized trial of a vaccine regimen to prevent chronic HCV infection. N. Engl. J. Med..

[120] Gomez-Escobar E., Roingeard P., Beaumont E. (2023). Current hepatitis C vaccine candidates based on the induction of neutralizing antibodies. Viruses.

[121] Rzymski P., Jibril A.T., Rahmah L., Abarikwu S.O., Hashem F., Lawati A.A., Morrison F.M.M., Marquez L.P., Mohamed K., Khan A. (2024). Is there still hope for the prophylactic hepatitis C vaccine? A review of different approaches. J. Med. Virol..

[122] Zemlin M., Klinger M., Link J., Zemlin C., Bauer K., Engler J.A., Schroeder H.W., Kirkham P.M. (2003). Expressed murine and human CDR-H3 intervals of equal length exhibit distinct repertoires that differ in their amino acid composition and predicted range of structures. J. Mol. Biol..

[123] Guo Y., Bao Y., Meng Q., Hu X., Meng Q., Ren L., Li N., Zhao Y. (2012). Immunoglobulin genomics in the guinea pig (*Cavia porcellus*). PLoS ONE.

[124] Abe K., Inchauspe G., Shikata T., Prince A.M. (1992). Three different patterns of hepatitis C virus infection in chimpanzees. Hepatology.

[125] Berggren K.A., Suzuki S., Ploss A. (2020). Animal models used in hepatitis C virus research. Int. J. Mol. Sci..

[126] Bartosch B., Bukh J., Meunier J.-C., Granier C., Engle R.E., Blackwelder W.C., Emerson S.U., Cosset F.-L., Purcell R.H. (2003). In vitro assay for neutralizing antibody to hepatitis C virus: Evidence for broadly conserved neutralization epitopes. Proc. Natl. Acad. Sci. USA.

[127] Major M.E., Mihalik K., Puig M., Rehermann B., Nascimbeni M., Rice C.M., Feinstone S.M. (2002). Previously infected and recovered chimpanzees exhibit rapid responses that control hepatitis C virus replication upon rechallenge. J. Virol..

[128] Liang T.J., Feld J.J., Cox A.L., Rice C.M. (2021). Controlled human infection model—Fast track to HCV vaccine?. N. Engl. J. Med..

[129] Alter H.J., Barnes E., Biondi M.J., Cox A.L., Eberts J.D., Feld J.J., Liang T.J., Morrison J., Rice C.M., Shoukry N.H. (2023). Joint statement in support of hepatitis C human challenge studies. Lancet Gastroenterol. Hepatol..

[130] Darton T.C., Blohmke C.J., Moorthy V.S., Altmann D.M., Hayden F.G., Clutterbuck E.A., Levine M.M., Hill A.V., Pollard A.J. (2015). Design, recruitment, and microbiological considerations in human challenge studies. Lancet Infect. Dis..

[131] Weiner A.J., Geysen H.M., Christopherson C., Hall J.E., Mason T.J., Saracco G., Bonino F., Crawford K., Marion C.D., Crawford K.A. (1992). Evidence for immune selection of hepatitis C virus (HCV) putative envelope glycoprotein variants: Potential role in chronic HCV infections. Proc. Natl. Acad. Sci. USA.

[132] Zibert A., Meisel H., Kraas W., Schulz A., Jung G., Roggendorf M. (1997). Early antibody response against hypervariable region 1 is associated with acute self-limiting infections of hepatitis C virus. Hepatology.

[133] Adhikari A., Eltahla A., Lloyd A.R., Rodrigo C., Agapiou D., Bull R.A., Tedla N. (2021). Optimisation and validation of a new method for antibody dependent cellular phagocytosis in hepatitis C virus infection. J. Immunol. Methods.

[134] Nattermann J., Schneiders A.M., Leifeld L., Langhans B., Schulz M., Inchauspé G., Matz B., Brackmann H.H., Houghton M., Sauerbruch T. (2005). Serum antibodies against the hepatitis C virus E2 protein mediate antibody-dependent cellular cytotoxicity (ADCC). J. Hepatol..

[135] Pihl A.F., Feng S., Offersgaard A., Alzua G.P., Augestad E.H., Mathiesen C.K., Jensen T.B., Krarup H., Law M., Prentoe J. (2022). Inactivated whole hepatitis C virus vaccine employing a licensed adjuvant elicits cross-genotype neutralizing antibodies in mice. J. Hepatol..

[136] Gridley J., Holland B., Salinas E., Trivedi S., Dravid P., Elrod E., Jin F., Kumari A., Batista M.N., Thapa M. (2024). Concerted synergy between viral-specific IgG and CD8+ T cells is critical for clearance of an HCV-related rodent hepacivirus. Hepatology.

